# Radiation dose reduction and improvement of image quality in digital chest radiography by new spatial noise reduction algorithm

**DOI:** 10.1371/journal.pone.0228609

**Published:** 2020-02-21

**Authors:** Wonje Lee, Seungho Lee, Semin Chong, Kyungmin Lee, Jongha Lee, Jae Chol Choi, Changwon Lim

**Affiliations:** 1 Clinical Research Group, Health & Medical Equipment Business, Samsung Electronics, Suwon, Korea; 2 Department of Radiology, Chung-Ang University Hospital, Seoul, Korea; 3 Department of Radiology, Samsung Medical Center, Sungkyunkwan University School of Medicine, Seoul, Korea; 4 Medical Imaging R&D Group, Health & Medical Equipment Business, Samsung Electronics, Suwon, Korea; 5 Division of Pulmonary Medicine, Department of Internal Medicine, Chung-Ang University College of Medicine, Chung-Ang University, Seoul, Korea; 6 Department of Applied Statistics, Chung-Ang University, Seoul, Korea; INSERM, FRANCE

## Abstract

**Purpose:**

To evaluate the image quality of low-dose chest digital radiographic images obtained with a new spatial noise reduction algorithm, compared to a conventional de-noising technique.

**Materials and methods:**

In 69 patients, the dose reduction protocol was divided into A, B, and C test groups– 60% (n = 22), 50% (n = 23), and 40% (n = 24) of the baseline dose. In each patient, baseline dose radiographs were obtained with conventional image processing while low-dose images were acquired with new image processing. A set of baseline and low-dose radiographic images per patient was evaluated and scored on a 5-point scale over seven anatomical landmarks (radiolucency of unobscured lung, pulmonary vascularity, trachea, edge of rib, heart border, intervertebral disc space, and pulmonary vessels in the retrocardiac area) and three representative abnormal findings (nodule, consolidation, and interstitial marking) by two thoracic radiologists. A comparison of paired baseline and low-dose images was statistically analyzed using a non-inferiority test based on the paired t-test or the Wilcoxon signed-rank test.

**Results:**

In A, B, and C test groups, the mean dose reduction rate of the baseline radiation dose was 63.4%, 53.9%, and 47.8%, respectively. In all test groups, the upper limit of the 95% confidence interval was less than the non-inferiority margin of 0.5 every seven anatomical landmarks and three representative abnormal findings, which suggested that the image quality of the low-dose image was not inferior to that of the baseline dose image even if the maximum average dose reduction rate was reduced to 47.8% of the baseline dose.

**Conclusion:**

In our study, an image processing technique integrating a new noise reduction algorithm achieved dose reductions of approximately half without compromising image quality for abnormal lung findings and anatomical landmarks seen on chest radiographs. This feature-preserving, noise reduction algorithm adopted in the proposed engine enables a lower radiation dose boundary for the sake of patient’s and radiography technologist’s radiation safety in routine clinical practice, in compliance with regulatory guidelines.

## Introduction

According to the European guidelines issued by the Commission of the European Communities (CEC), chest radiography radiation dose criteria are 0.3 mGy based on the entrance surface dose for a standard-sized patient. Currently, most chest radiographic equipment meets these requirements [1]. However, in terms of the ALARA (As Low As Reasonably Achievable) principle used in radiation safety, it remains a challenge to continually reduce the radiation dose of chest radiography. In particular, the task is to reduce the radiation dose of chest radiography without sacrificing diagnostic confidence [2]. In digital radiography, a multi-faceted approach to hardware improvement or software development has made it possible to lower radiation doses [3].

In a retrospective study of digital chest radiography, Grewal et al. reported that by utilizing additional filtration adding to conventional requirements (at least 2.5 mm Al equivalent), the calculated effective dose (mSv) was reduced by 52% without compromising image quality [4]. On the other hand, there have been reports that advanced image processing can also affect dose reduction by providing optimal imaging parameters. Among them, multiscale frequency processing algorithms have been applied to digital chest radiography, improving the low-density structure and achieving better subtle pathological conditions [5–7]. However, those approaches may not provide optimum noise reduction in the region of locally varying imaging features. Since bones, soft tissues, and/or subtle regions of the disease have a different boundary and signal intensity, estimating precise noise distributions may need to take such spatially varying imaging features into account. Recently, we have devised a new spatially-adaptive noise reduction algorithm based on multi-scale noise covariance, including multi-scale frequency processing with a non-local mean method and noise whitening technique.

In our previous experimental study using chest phantoms, we hypothesized that this new spatial noise reduction algorithm capable of effectively reducing noise while preserving organ and vascular boundaries over a multi-scale noise covariance processing algorithm would improve the quality of chest x-ray images acquired at low doses. By applying our proposed spatial noise reduction algorithm, it was found that the overall phantom image quality can be improved in low dose chest radiographs of both anatomical and anthropomorphic chest phantoms [S1 Appendix]. Therefore, the purpose of this study was to evaluate the image quality of low-dose digital chest radiography obtained from the human body using this new spatial noise reduction algorithm.

## Materials and methods

The Institutional Review Board of Chung-Ang University Hospital approved this prospective study (IRB No.1601-012-255) and written informed consent was obtained for all subjects included in this study. The participants agreed to publish their radiographs and completed an informed consent form for publication. From January 2, 2017 to January 31, 2017, a pair of chest posteroanterior (PA) radiographs with standard and reduced radiation dose was obtained in patients who had either initial or follow-up chest PA examinations for the evaluation of diagnosis and treatment while visiting the Department of Pulmonology at the institution. The inclusion criteria for this study were adults over 20 years of age with a body mass index (BMI) of 18.5–29.9 kg/m^2^ who agreed to voluntarily participate in a chest X-ray examination. Exclusion criteria were females who were pregnant, currently breastfeeding, or who expected to be pregnant within a month after participation. Finally, a total of 69 subjects were enrolled in this study. The dose reduction protocol was divided into A, B, and C test groups in a blind fashion– 60% (n = 22), 50% (n = 23) and 40% (n = 24) of the baseline dose, respectively.

**Fig 1:**
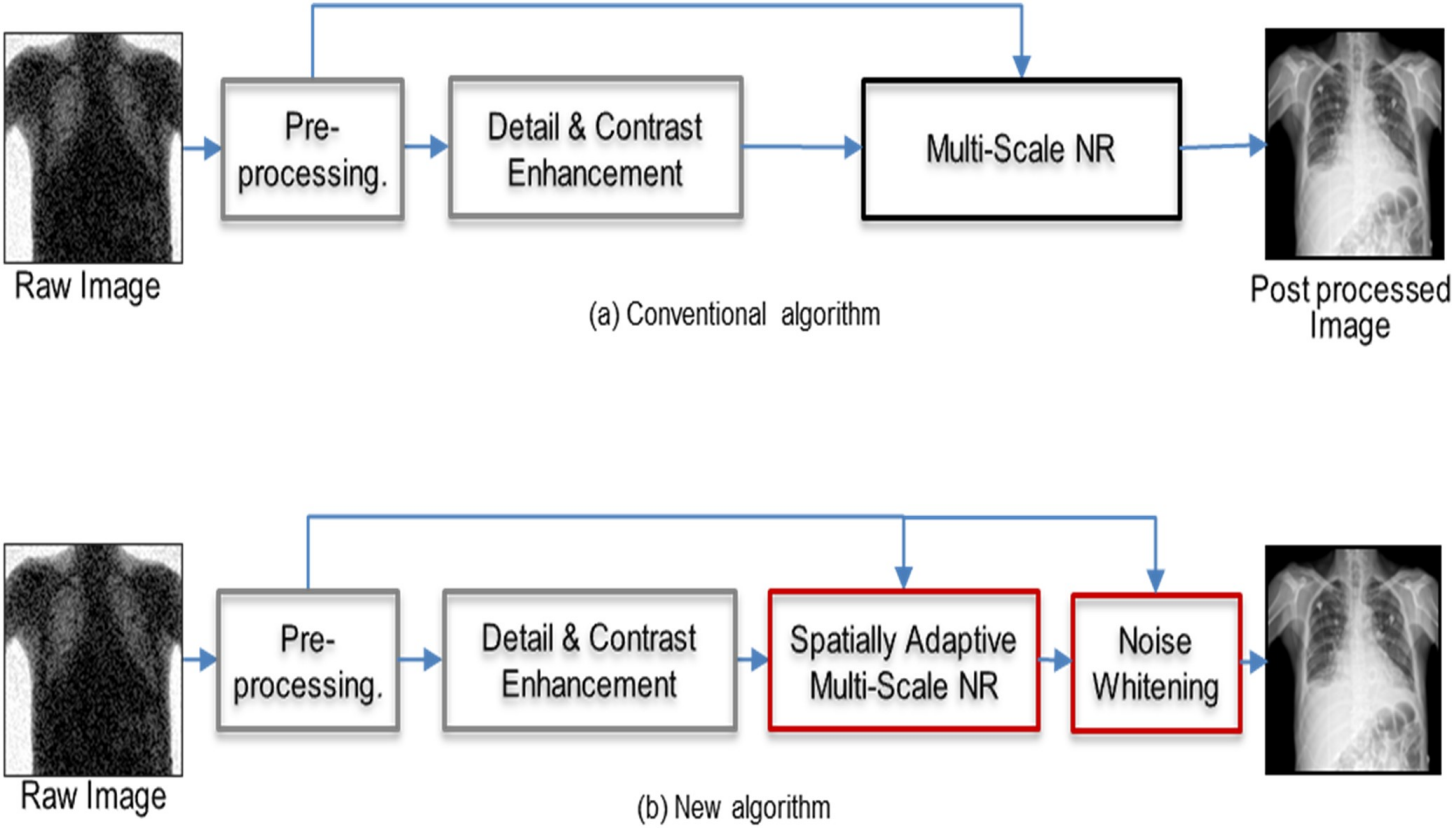
A schematic diagram comparing the conventional (a) and new algorithms (b).

Each patient took two digital chest radiographs with two consecutive X-ray exposures (GC85A, Samsung Electronics, Suwon, Korea) on the same day—one for the baseline dose and the other for the reduced dose. The baseline radiation dose was controlled with a cutoff value of 4.2 μGy in the use of automatic exposure control (AEC), which is measured on the detector surface, over which the exposure control automatically shuts down the X-ray generator and determined by the routine chest PA exposure of the institution. The low-dose levels relative to the baseline were controlled by discrete AEC steps, and the most closely corresponding AEC cutoff values were 2.5 μGy, 2.11 μGy, and 1.78 μGy for each reduced dose of the A, B, and C groups respectively. The imaging acquisition parameters of the digital X-ray machine for all chest PA radiographs were as follows: filter, 0.1 mm Cu; source to image distance (SID), 180 cm; grid focal distance, 1,800 mm; and focal spot size, 1.2 mm. Entrance skin exposure (ESE) was calculated for the given mAs and kVp and the calibrated source to object distance (SOD) to the patient thickness for each subject based on the pre-defined tube output of the X-ray system. Baseline radiographs were obtained by applying a conventional image processing engine (S-Vue^™^ 3.00, Samsung Electronics, Suwon, Korea) on an image processing workstation attached to an X-ray machine. To apply a new image processing engine (S-Vue^™^ 3.02, Samsung, Suwon, Korea), raw data from chest radiographs taken at reduced doses was transferred to a dedicated workstation to acquire low-capacity images. We have devised this new spatially-adaptive noise reduction algorithm based on multi-scale noise covariance, including multi-scale frequency processing with a non-local mean method and noise whitening technique [7]. Multi-scale noise covariance well reflects spatially dependent noise distribution, mainly consisting of scatter and blur noises attributed to the interaction of photons with an imaging object and signal conversion process within a detector (Fig 1). Spatially adaptive multi-scale process accommodated spatially-varying local image features to handle region-specific noise more precisely in order to preserve local boundary information. This process allowed to have minimal edge information lost, however, inevitably produced coarse noise that entailed degrading observer performance. To handle coarse noise, a noise whitening process was added in order to de-correlate pixel noise, enhancing visual performance. Both multi-scale noise reduction and noise whitening block utilized a noise map extracted from pre-processed images. These two differentiated function block synergistically contributed to improving image quality at low dose exposures.

A set of baseline and low-dose chest radiographic images per patient was evaluated twice in a blinded fashion by two radiologists with 2 and 20 years of experience in thoracic radiology. To clear the memory of the first reading session, there was a two week time interval between the two reading sessions. Both 69 baseline images and 69 low-dose images, a total of 138 images, were randomly numbered from No.1 to No.138 in advance and scored one by one on a 5-point scale for seven anatomical landmarks (determined by reference to the Korean Institute for Accreditation of Medical Imaging) and three representative abnormal radiographic findings using the Likert scale without a reference image (1, not relevant for diagnostic images; 2, poor image quality; 3, fair image quality; 4, good image quality; and 5, excellent image quality) under the same reading conditions (Table 1). The scores of the seven anatomical landmarks were summed up to give a total score of 35. In 28 of 69 subjects, three kinds of representative abnormal radiographic findings were selected by another radiologist with 13 years of experience in thoracic radiology, which included 12 nodules, 13 consolidations, and 15 interstitial markings.

**Table 1:** Evaluation criteria for chest radiographic images.

Anatomical landmarks	
AL1	Radiolucency of unobscured lung
AL2	Pulmonary vascularity
AL3	Trachea
AL4	Edge of rib
AL5	Heart border
AL6	Intervertebral disc space
AL7	Pulmonary vessel in the retrocardiac area
AL total	Sum of the score of seven anatomical landmarks
Representative abnormal radiographic findings	
ND	Nodule
CD	Consolidation
IST	Interstitial marking

AL = anatomical landmarks

Between the A, B and C groups, one-way ANOVA with a Tukey post hoc test was used to verify the uniformity of the subject’s BMI and baseline radiation dose, differentiation of dose reduction rates, and homogeneity of either baseline or low-dose image quality. Across 69 patients, a total of 138 images were analyzed using an average score that was evaluated twice by two radiologists for anatomical landmarks and abnormal findings of the chest radiograph. Inter-observer agreement between two radiologists was quantified by the intraclass correlation coefficient (ICC), of which the ICC value was interpreted as follows: < 0.40, poor; 0.40–0.59, fair; 0.60–0.74, good; and 0.75–1.00, excellent. The comparison between the paired baseline and low-dose images of each patient was statistically analyzed using a non-inferiority test as follows. The upper bound of the 95% confidence interval of a mean difference (paired t-test) in quality scales between the baseline and low-dose images was calculated to confirm non-inferiority. The quality scale at the low-dose was assumed to be statistically non-inferior to its scale at baseline dose if the upper bound of the 95% confidence interval of a mean difference is smaller than the non-inferiority margin of 0.5 for each of seven anatomical landmark scores and 3.5 (0.5 x 7) margin for the total score. The three representative abnormal radiographic findings (nodule, consolidation, and interstitial marking) were also analyzed by applying the non-inferior margin of 0.5. The Shapiro-Wilk test was performed on the normality test. For pairwise comparison of baseline and low-dose images, a paired t-test was used when normality was assumed, and the Wilcoxon signed-rank test was used for the case of not assuming normality. Statistical analysis was performed using MedCalc (version 18.11.6, MedCalc Software) and IBM SPSS Statistics for Windows, Version 21.0 (IBM Corp., Armonk, NY, USA). A p-value < 0.05 was considered statistically significant.

## Results

Table 2 shows a summary of BMI and radiation dose information in each group for the 69 patients. There were no significant differences in BMI and baseline radiation dose among the three groups (p = 0.079 and p = 0.340, respectively). This means that there was no selection bias between the groups because the BMI of the subject was constant and the baseline dose was homogeneous. In all 69 patients, the dose level was significantly different between baseline and reduced dose sets (p < 0.001). In addition, there was a statistically significant difference of dose reduction rate between all groups in the multi-group comparison of dose reduction rates (A vs. B, B vs. C and C vs. A) (p < 0.001), reflecting that there was a differentiated dose reduction between the groups.

**Table 2:** Summary of BMI and radiation dose information in 69 subjects.

	BMI (kg/m^2^)	Baseline dose (ESE, μGy)	Low dose (ESE, μGy)	Reduced dose rate (%)^a^
Total (n = 69)	23.9 ± 2.4	47.0 ± 9.8	25.7± 5.9	54.8 ± 6.7 (43.1–66.1)
Group A (n = 22)	23.1 ± 2.2	47.3 ± 10.2	29.9 ± 6.1	63.4 ± 1.6 (60.7–66.1)
Group B (n = 23)	23.9 ± 2.4	44.7 ± 7.8	24.1 ± 4.5	53.9 ± 1.9 (50.8–58.7)
Group C (n = 24)	24.7 ± 2.4	48.9 ± 11.1	23.3 ± 4.7	47.8 ± 2.0 (43.1–50.4)

BMI = body mass index ESE = entrance skin exposure

^a^ means the percentage of baseline dose. All numbers are mean ± standard deviation and the number in parentheses is range.

**Fig 2:**
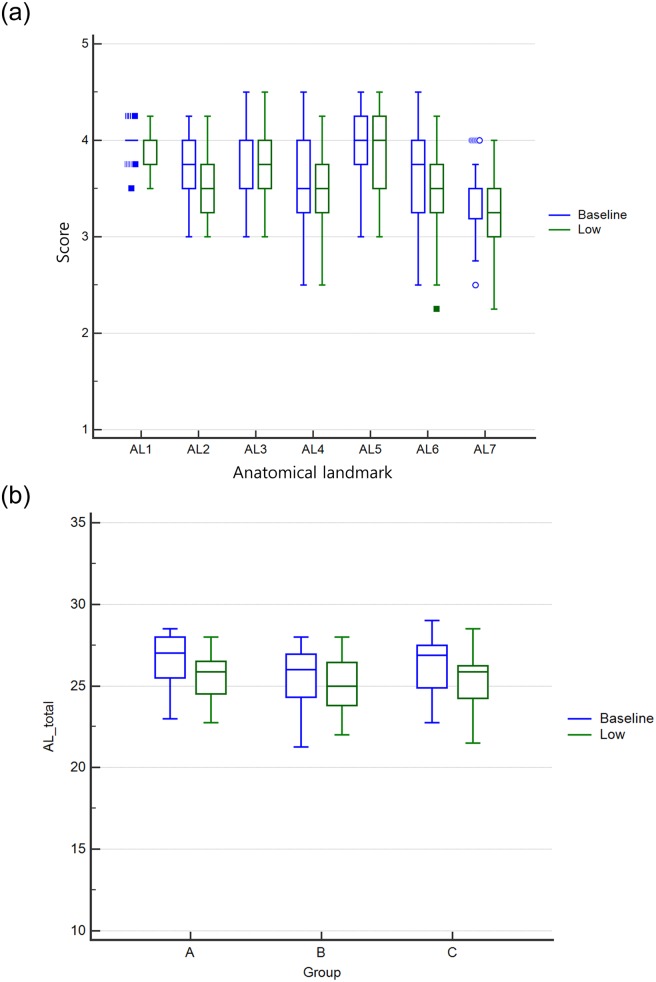
Box-whisker plots of each score of seven anatomical landmarks in all 69 patients (a) and total scores of seven anatomical landmarks in three test groups (b) according to radiation dose. The out and far-out data are presented as circular and rectangular symbols, respectively.

According to the inter-observer agreement, the calculated ICC between two radiologists was 0.747 in a total of 138 images, 0.796 in 69 baseline images, and 0.704 in 69 low-dose images, which was fair or good. In both baseline and low-dose chest radiographic images of all 69 patients, the minimum score of each anatomical landmark was 3 or more, except for AL4 (edge of rib), AL6 (intervertebral disc space), and AL7 (pulmonary vessel in the retrocardiac area) (Fig 2a). However, the lower (first) quartile of each anatomical landmark score was all above a score of 3, suggesting fair image quality even in low-dose images as well as baseline images while that of the total score of the seven anatomical landmarks was all above 21, which was also suggestive of fair image quality on average (Fig 2b). Between the A, B, and C groups, there were no significant differences in seven anatomical landmark scores (AL1~AL7) of either baseline or low-dose images (AL1, p = 0.108 and p = 0.901; AL2, p = 0.167 and p = 0.171; AL3, p = 0.784 and p = 0.726; AL4, p = 0.183 and p = 0.453; AL5, p = 0.275 and p = 0.724; AL6, p = 0.101 and p = 0.191; AL7, p = 0.241 and p = 0.126, respectively). Between the A, B, and C groups, there were no significant differences in total score (AL total) of either baseline or low-dose images (p = 0.095 and p = 0.401, respectively). This means that all images to be evaluated had homogeneous image quality among the three groups and that there was no image selection bias.

**Fig 3:**
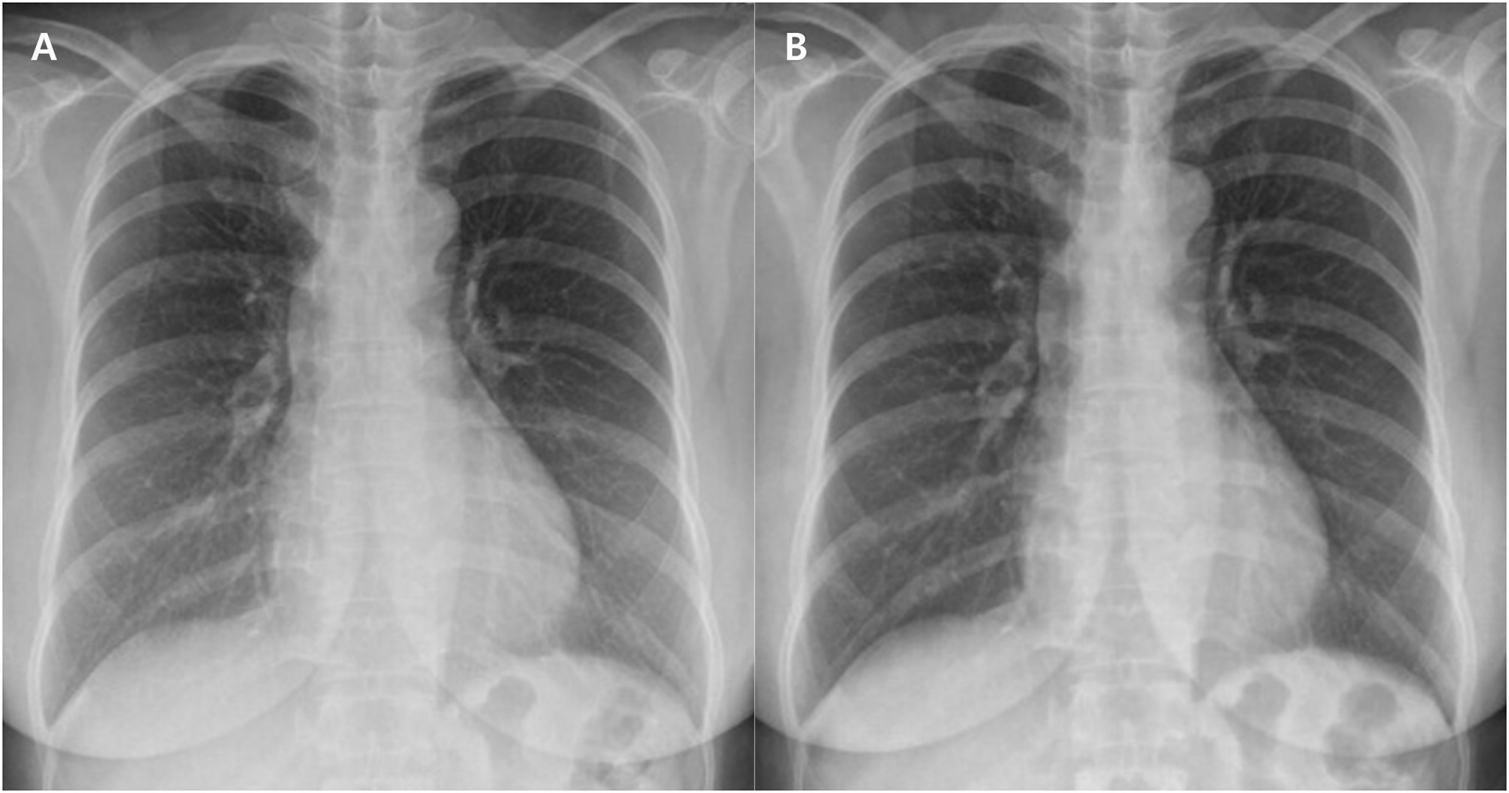
A pair of baseline (a) and low-dose (b) chest radiographic images in a patient with a BMI of 23.7 kg/m^2^ (Group C). The radiation dose of ESE was 42.9 μGy and 21.2 μGy respectively, which was reduced to 49.4% of the baseline dose. The average total score of anatomical landmarks was 26.8 and 28.5 respectively. The image quality of the low-dose image was non-inferior and superior to that of the baseline dose image.

Table 3 summarizes the non-inferiority test results of anatomic landmarks between the paired baseline and low-dose images in the total group, group A, group B, and group C. In all test groups, the upper limit of the 95% confidence interval was less than the non-inferiority margin of 0.5 every seven anatomical landmarks, and less than 3.5 for the total of the anatomical landmarks, which means that the image quality of the low-dose image was not inferior to that of the baseline dose image even if the maximum average dose reduction rate was reduced to 47.8% of the baseline dose (Fig 3).

**Table 3:** Comparison of image quality of anatomical landmarks between the baseline and low-dose images using a non-inferiority test in total, A, B, and C groups.

Group	Total			Group A			Group B			Group C		
Evaluation	Mean difference	95% CI		Median difference	95% CI		Mean difference	95% CI		Median difference	95% CI	
		Lower	Upper		Lower	Upper		Lower	Upper		Lower	Upper
AL 1	0.069	0.022	0.116	0.000	0.000	0.125	0.000 ^a^	0.000 ^a^	0.125 ^a^	0.125	0.000	0.125
AL 2	0.145	0.076	0.214	0.125	0.000	0.250	0.130	-0.011	0.272	0.125	0.000	0.250
AL 3	0.047	-0.026	0.120	0.000	0.000	0.125	0.011	-0.133	0.155	0.063 ^b^	-0.078 ^b^	0.203 ^b^
AL 4	0.098	0.026	0.169	0.000	0.000	0.125	0.065	-0.066	0.196	0.125	0.000	0.250
AL 5	0.112	0.041	0.183	0.125	0.000	0.250	0.000 ^a^	-0.125 ^a^	0.250 ^a^	0.125	0.000	0.250
AL 6	0.145	0.080	0.210	0.125	0.000	0.250	0.125 ^a^	0.000 ^a^	0.250 ^a^	0.125	0.000	0.250
AL 7	0.138	0.055	0.220	0.125	0.000	0.250	0.022	-0.104	0.148	0.250	0.125	0.500
AL total	0.754	0.495	1.012	0.750	0.375	1.125	0.446	-0.038	0.929	1.250	0.625	1.500

CI = confidence interval AL = anatomical landmarks

The quality scale at the low dose was assumed to be statistically non-inferior to its scale at baseline dose if the upper bound of 95% confidence interval of a mean difference is smaller than non-inferiority margin of 0.5 for each of seven anatomical landmark scores and 3.5 (0.5 x 7) margin for the total score.

^a^ was described as median difference because normality was not assumed, and

^b^ was described as mean difference because normality was assumed.

**Fig 4:**
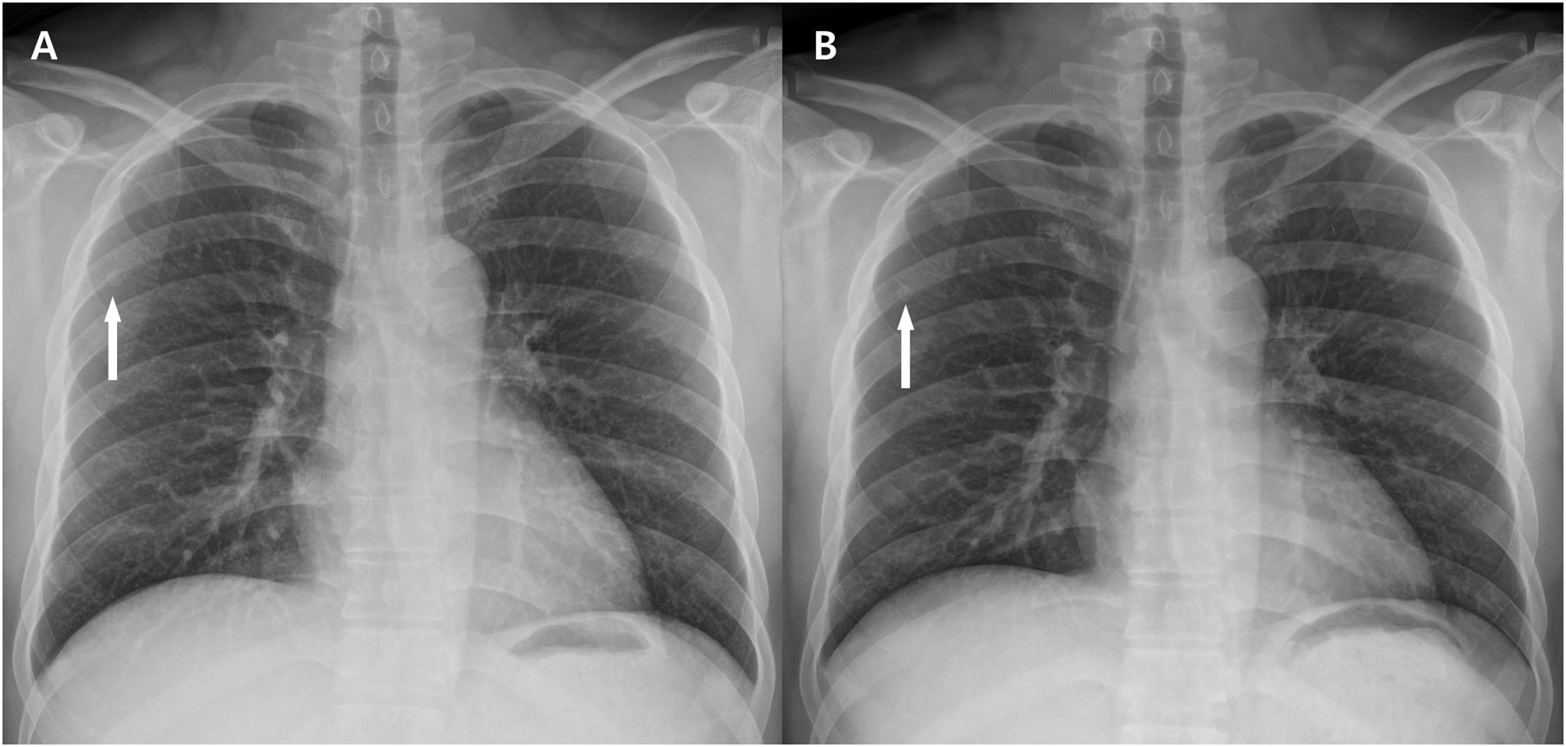
A pair of baseline (a) and low-dose (b) chest radiographic images in a patient with a BMI of 22.8 kg/m^2^ (Group C). The two chest radiographs show a well-defined, small nodule in the right upper lung zone (arrows). The average score of the nodule was 4 and 3.75 respectively, which were suggestive of good image quality. The radiation dose of ESE was 61.9 μGy and 30.2 μGy respectively, which was reduced to 48.8% of the baseline dose.

**Fig 5:**
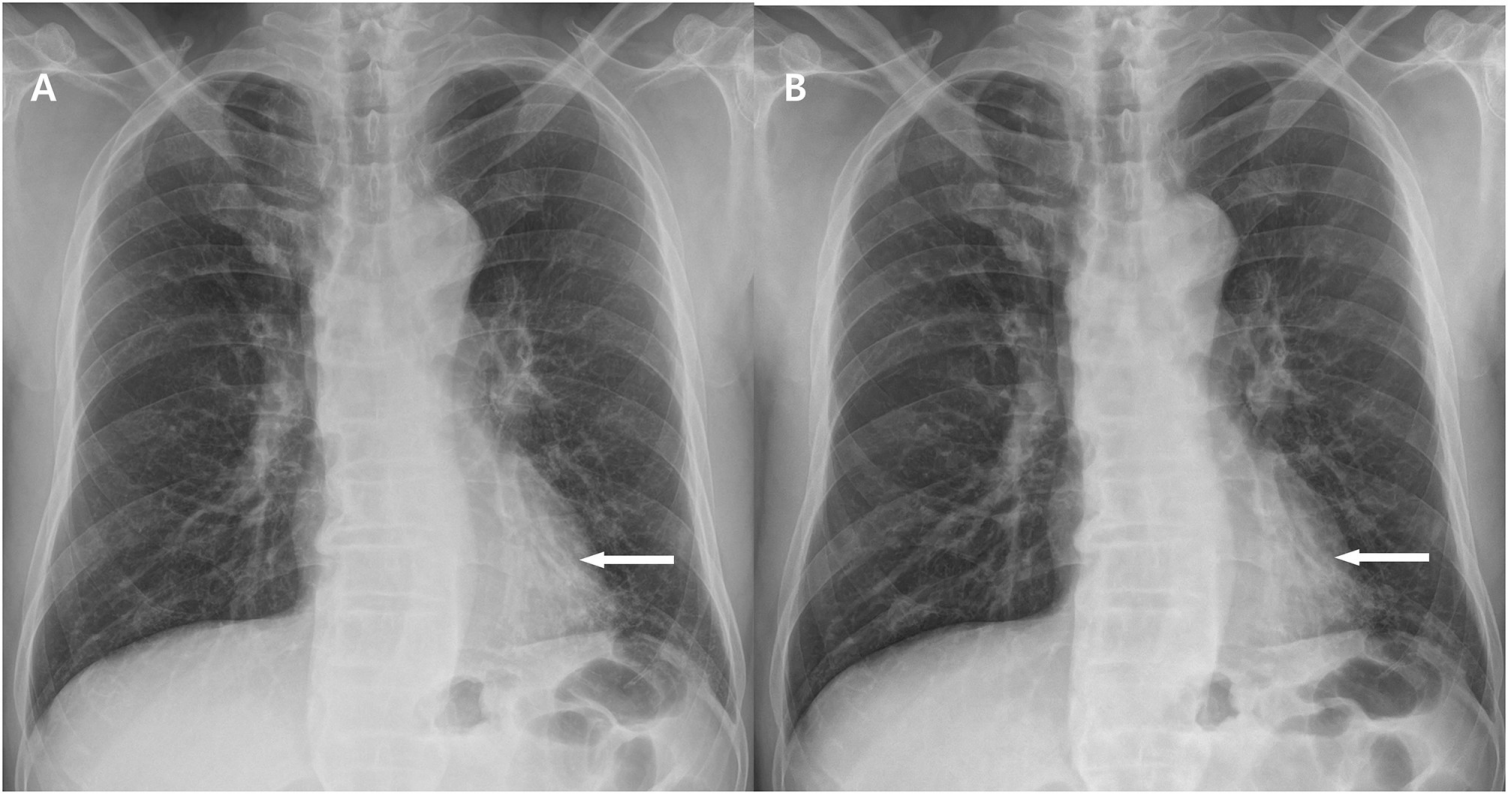
A pair of baseline (a) and low-dose (b) chest radiographic images in a patient with a BMI of 25.4 kg/m^2^ (Group C). The two chest radiographs show a subsegmental consolidation in a retrocardiac region of a left lower lung zone (arrows). The average score of the consolidation was 3.50 and 3.00 respectively, which were suggestive of fair image quality. The radiation dose of ESE was 54.2 μGy and 25.0 μGy respectively, which was reduced to 46.1% of the baseline dose.

Table 4 shows the non-inferiority test results between the paired baseline and low-dose images for three representative abnormal radiographic findings in 28 subjects. For all test findings, the upper limit of the 95% confidence interval was less than the non-inferiority margin of 0.5, which means that the image quality of the low-dose image was not inferior to that of the baseline dose image (Figs 4 and 5).

**Table 4:** Comparison of image quality of three representative abnormal findings between the baseline and low-dose images using a non-inferiority test.

Findings	Mean		Median		Mean difference	95% CI	
	Baseline	Low dose	Baseline	Low dose		Lower	Upper
ND	3.208	3.042	3.000	3.000	0.167	-0.004	0.337
CD	3.212	3.135	3.250	3.000	0.000 ^a^	0.000 ^a^	0.250 ^a^
IST	3.133	2.833	3.250	2.750	0.250 ^a^	0.125 ^a^	0.500 ^a^

CI = confidence interval ND = Nodule CD = consolidation IST = Interstitial marking

The quality scale at the low dose was assumed to be statistically non-inferior to its scale at baseline dose if the upper bound of 95% confidence interval of a mean difference is smaller than the non-inferiority margin of 0.5 for each abnormal finding.

^a^ was described as a median difference because normality was not assumed.

## Discussion

A uniform cut-off reduction rate cannot be applied to a number of clinical sites because each site sets a different standard dose level with the preferred image at its own discretion. However, in many clinical settings, despite these subjective settings, the ALARA principle must be observed in two major aspects of the radiation safety regulatory guidelines which are “optimization” and “justification” [8]. In our dose reduction study of digital chest radiography to address this challenge, average dose reduction by 47.8% of the original dose was achieved in chest radiography without degradation of image quality by applying a new post-processing imaging algorithm.

Dose optimization is a recommended practice to emphasize the benefits of radiographic procedures by minimizing cumulative radiation risk given the trend of increasing demand for diagnostic imaging examinations. Multiple approaches are required to achieve this goal in the digital imaging era as a number of systematic factors are available to optimize imaging parameters. In the use of additional Cu filtration, an entrance skin dose was reduced down from 25% to 44% depending on the Cu filter thickness without compromising image quality [9]. The use of supplemental Cu filters benefits superficial sensitive organs in terms of effective dose. For instance, in the use of a Cu filter with an optimized tube voltage the breast tissue is less exposed by getting rid of low energy photons in the chest anteroposterior position (AP), helping to reduce the dose in pediatric chest examinations. In another method, the use of CsI/FPD is reported to be a most efficient detector in a tradeoff between dose level and image quality, while producing satisfactory diagnostic imaging at a relatively lower dose [10]. Quality assurance activity on a particular protocol also can lead to a noticeable dose reduction by means of optimizing tube voltage and current [11]. These activities are generally used as methods for enhancing dose reduction in digital radiography.

The aforementioned dose optimization practice is only meaningful when diagnostic performance is not compromised due to lowering dose levels. An observer study that evaluated the image quality of digital chest radiographs at different dose levels using low-dose simulation suggested that a dose reduction in half seems feasible in a variety of chest pathologies, indicating no significant loss in diagnostic performance with a half dose [12]. On the other hand, image noise attributed to lowering dose seems to affect detection performance depending on the location of the lesion [13]. In lung fields, there is no indication of significant effects on reading performance, but in the mediastinum area, there is a reported noticeable influence of image noise-to-nodule detection performance. This study indicates that noise distributions are spatially dependent within an image, necessitating reliable noise estimation and reduction to compensate for added noise due to low-dose setup. In our study, the lowest score of AL6 (intervertebral disc space) and AL7 (pulmonary vessel in the retrocardiac area), which were anatomical landmarks of mediastinal and low contrast areas, was 2 points and showed poor image quality at the baseline dose. However, the new image processing algorithm seems to suppress degradation of the image quality at a low dose because the low 25% score was not less than 3 points (fair image quality) as well as the non-inferiority compared to the baseline dose image.

In digital imaging post-processing, many existing noise reduction methods rely on information around adjacent pixels or local area to estimate denoised pixel intensity, known as a local-based spatial method [14–16]. A non-local image noise algorithm developed by Buades et al. reported superior performance, attributed to the utilization of image redundancy [17]. Liu et al. introduced a further developed algorithm to expedite the calculation process based on non-local similarity in frequency components [18]. Those special noise filters successfully demonstrated denoising performance however they concomitantly produced correlated noises, deteriorating visual context. The advanced denoising algorithm applied in our study was designed to incorporate a noise decorrelation technique along with a non-local mean algorithm, sustaining noise suppression performance without degrading visual context. This hypothesis could be proved through the results of this study that the image quality of the low-dose image was not inferior to that of the conventional dose image when the new denoising algorithm was applied to the image where the degradation of the image quality was predicted. Successful noise reduction performance produced non-inferior and equivalent image qualities over various anatomical landmarks even under half doses.

Our study has several limitations. First, our study included a relatively small number of subjects. Assuming a significance level of 0.025, power of 0.8, and a standard deviation of 0.49, the minimum number of samples in each group was predicted to be 24. However, as a result of applying inclusion and exclusion criteria, two and one patients were insufficient in A and B groups, respectively. However, we focused on comparing a pair of baseline and low-dose images taken at nearly the same time in the same patient, and therefore it was optimized for meaningful pairwise comparison studies. Second, our study population did not have diverse abnormal findings because most patients were initially referred to the hospital or, even if they were followed up, the presence or absence of the lesion could not be predicted. Third, our study included subjects with only normal and overweight BMIs to minimize bias to BMI, which limited the evaluation of underweight or obese patients. In the future, a large-scale prospective study is needed. Fourth, the performance of our proposed algorithm may be dependent on vendor-specific systems. In general, this algorithm can be applied to most digital radiographs, but system compatibility, including detector type and prerequisites for raw data, should be investigated before applying it to other systems.

## Conclusions

In conclusion, our study suggests that the image processing technique integrating a new noise reduction algorithm could achieve dose reductions approximately by half without compromising the image quality of abnormal lung findings as well as anatomical landmarks seen on chest radiographs. Although our study was conducted at a single clinical site, covering a limited number of subjects with a single protocol, the application of the new noise reduction algorithm can be expanded to other clinical protocols since its application is not bounded to a particular protocol. We believe that the feature-preserving and noise reduction algorithm adopted in the proposed engine enables decreasing a lower dose bound for the sake of patients and radiography technologist’s radiation safety in routine clinical practices.

## Supporting information

S1 Appendix Supplemental material for our noise reduction algorithm. (DOCX)
