# Supplementary material for: Radiation dose reduction and improvement of image quality in digital chest radiography by new spatial noise reduction algorithm
Source: PLoS One. 2020 Feb 21;15(2):e0228609. doi: 10.1371/journal.pone.0228609 (PMC7034827; doi:10.1371/journal.pone.0228609)
Supplement: S1 Appendix — (DOCX) [file pone.0228609.s001.docx]

**Supplemental Material for Our Noise Reduction Algorithm**

1. **Introduction**

Our proposed algorithm for low dose image improvement is about advanced noise redirection technology, which solves image quality degradation problems with increasing noise in low dose. The algorithm consists of three main blocks. Each function block effectively predicts noise, suppresses the predicted noise, and improves the visual performance of clinical X-ray images.

***Noise Estimation***

Our proposed noise estimation technology is based on a signal-dependent noise covariance instead of noise variance currently used [1]. Noise in X-ray images consists mainly of Poisson noise in the X-ray generation process, scatter noise derived from the interaction process between object and x-ray photons, and electric noise occurred in X-ray detector. Under low-dose conditions, the combination of the above noises generates the low-frequency component noise of the coarse-grain type, which significantly affects the degradation of X-ray image quality. We found that the signal-dependent noise covariance is a good model that can describe spatially-dependent noise. Based on this finding, we predict noise distribution for noise reduction.

***Noise Filtering***

Noise filtering is a process of removing noise by utilizing spatially-adjacent pixel information for a single image. Our noise reduction filter is based on the Non-Local Mean (NLM) method [2, 3], which is known as a state-of-the-art technique among the analytical noise reduction methods. The NLM-based filtering method reduces image noise by finding similar patches around the reference patches within a defined field of view (FOV) and summing them with proper weights. In order to find similar patches around them and to apply weighted-summation for them, a precisely-estimated noise map information and determination of their weights are required.

We implemented the noise reduction method without loss of structural information of the original signal and determination of the correct NLM weights by accurately predicting the noise characteristics in the low dose condition.

***Noise Whitening***

Even though noise is reduced through our noise filtering, there is a limit to removing coarse-grained noise with low-frequency characteristics. Compared to high-frequency noise, the coarse-grained noise of low-frequency components is known to degrade the image visibility and the preference of radiologists who read X-ray images of images [4]. In order to improve the image visibility, we transformed the low frequency component (coarse-grained type) noise to the white noise of fine-grained type, which is the noise characteristic of standard-dose images.

Noise estimating, filtering, and noise texture enhancing algorithms constitute the core of the advanced denoising algorithm. It does not only enhance the image quality itself but also generate clinical image that radiologists most prefer without degradation of clinical information. Therefore, the purpose of the following preclinical experiment study is to investigate the feasibility of our noise reduction algorithm by evaluating image quality of low-dose digital chest radiographs of semi-anatomical and anthropomorphic chest phantoms before attempting to perform very low-dose chest radiography on humans.

1. **Preclinical experiment of radiation dose reduction on digital chest radiography applied by noise reduction algorithm using semi-anatomical and anthropomorphic chest phantoms**

The institutional review board (IRB) approved a waiver of the IRB requirements for this phantom study because it was not a human subject study and did not even cause a minimum risk to the subject (IRB No.1802–003–16144).

We obtained the digital chest radiographic images using two types of phantoms, a) a semi-anatomical digital chest phantom (Duke 07–646, Supertech, IN, USA) for quantitative image analysis and b) an anthropomorphic chest phantom (N1 Lungman, Kyoto Kagaku Co., Ltd., Kyoto, Japan) for quantitative image analyses. Digital chest radiography (GC85A, Samsung Electronics, Suwon, Korea) was performed with the aforementioned phantoms using the following conditions: posteroanterior projection, standing position, medium size, focused grid, 0.1 mm copper filter = 0.1 mm, source-to-image-receptor distance (SID) = 180 cm, and Bucky-detector gap = 26 mm. For image acquisition, the raw data of chest radiography were processed with two different noise reduction algorithms; a conventional method (V_con_), and a new noise reduction algorithm (V_new_). We have designed a novel noise reduction algorithm, called a spatial noise reduction algorithm (V_new_), which was designed to adopt a spatially variable multiscale architecture that adjusted the degree of noise reduction with the use of nonlocal means depending on local image features. After noise filtration, a noise whitening technique was used for better noise texture restoration without compromising image quality. For instance, in the region of the edge, V_new_ led to a smaller degree of noise reduction and conserved boundary information, while in uniform homogeneous regions, it achieved a higher degree of noise reduction.

***Quantitative analysis using a semi-anatomical digital chest phantom (Duke phantom)***

We conducted experiments with the Duke phantom for objective assessments at the doses of 100%, 50%, 31%, 25%, 15%, and 8%, to the original dose (130 µGy, entrance skin exposure (ESE)). This phantom contains multiple regions-of-interest (ROIs) embedded in the high- and low-contrast areas. There were seven locations of ROIs in the high-contrast area, including five locations in the lung, two locations in the heart, and one location in the subphrenic area. In the low-contrast area, there were five ROIs, one in each of the lung fields, three in the heart, and one in the subphrenic area. We measured the signal-to-noise ratio (SNR) and contrast-to-noise ratio (CNR) in the ROIs according to the following equations.

***μ*** : mean of ROI

***σ* ^2^**: variance of ROI

***s_i_*** : # of ROI, ***i*** =1, 2

$$\boldsymbol{CNR=}\frac{\boldsymbol{|}\boldsymbol{\mu}_{\boldsymbol{s}\boldsymbol{1}}\boldsymbol{-}\boldsymbol{\mu}_{\boldsymbol{s}\boldsymbol{2}}\boldsymbol{|}}{\sqrt{\frac{{\boldsymbol{\sigma}_{\boldsymbol{s}\boldsymbol{1}}}^{\boldsymbol{2}}\boldsymbol{+}{\boldsymbol{\sigma}_{\boldsymbol{s}\boldsymbol{2}}}^{\boldsymbol{2}}}{\boldsymbol{2}}}}$$

***Qualitative analysis using an anthropomorphic chest phantom (Lungman phantom)***

We obtained images with the Lungman phantom for qualitative assessments at the doses of 100%, 50%, 31%, 25%, 15%, and 8%, to the original dose (130 µGy, ESE). The original dose (130 µGy) was determined based on the consideration of typical posterior-anterior (PA) chest exposures and image quality saturation within a range of exposure levels. The image quality of chest radiographs was scored twice by two thoracic radiologists with two years and 13 years of experience, respectively. The radiologists were blind to dose information and the imaging processing algorithm. They scored the images based on a ranked Likert scale using a reference by the modified Bureau of Radiological Health (BRH) scoring system (Table 1). The original BRH method consists of anatomic landmarks and physical parameters of 100 points out of a total of 200 points. Anatomical landmarks included the bony thorax, retrocardiac area, diaphragm, trachea, and pulmonary vasculature, which were scored based on the quality of visualization in chest radiographs. In addition, physical parameters included contrast, graininess, density, and detail [5]. In this study, we modified the original BRH method for the following reasons. The evaluation of the left diaphragm was excluded from our study because its outline was clearly defined from the time of production. Therefore, except for the score of the left diaphragm (15 points), the subtotal score of all the other anatomic landmarks was 85 points, and the final total score was 185 points (Table 1).

**Table 1. Modified Bureau of Radiological Health (BRH) scoring system.**

| **Anatomic landmarks (AL) (85)** | | | | |
| --- | --- | --- | --- | --- |
| Bony thorax (10)  (ribs & clavicles) | | Cortical margins (5) | AL1 | ( ) optimally visualized (5)  ( ) adequately visualized (4)  ( ) poorly visualized (2) ( ) not visualized (0)  ( ) disease precludes evaluation (N) |
|  |  | Trabeculae (5) | AL2 | ( ) optimal detail (5)  ( ) adequate detail (4)  ( ) poor detail (2)  ( ) no visualized (0)  ( ) disease precludes evaluation (N) |
| Retrocardiac area (15) | | Spine (15) | AL3 | ( ) too well visualized (7)  ( ) optimally visualized (15)  ( ) acceptably visualized (7)  ( ) poorly visualized (3) ( ) not visualized (0)  ( ) disease precludes evaluation (N) |
| Trachea (15) | | Visible to: (15) | AL4 | Visible to: (15)  ( ) left main stem bronchus (15)  ( ) carina (10)  ( ) neck and upper mediastinum (5)  ( ) not visible  ( ) disease precludes evaluation (N) |
| Diaphragm (15) | | Diaphragm outline (15) | AL5 | ( ) both visualized (15)  ( ) right only (7)  ( ) left only (7)  ( ) not visualized (0)  ( ) disease precludes evaluation (N) |
| Pulmonary vasculature (30) | Maximum measurable to: (30) | | AL6 | Maximum measurable to: (30)  ( ) right costophrenic angle (30)  ( ) right mid-lung (20)  ( ) right descending pulmonary artery (10)  ( ) none (0)  ( ) disease precludes evaluation (N) |
| **Physical parameters (PP) (100)** | | | | |
| Contrast (35) | | | PP1 | ( ) optimal (35)  ( ) good (23)  Poor, but diagnostic  ( ) too gray (11)  ( ) too black/white (11)  Unacceptable not diagnostic  ( ) too gray (0)  ( ) too black/white (0) |
| Density (15) | | | PP2 | ( ) optimal (15)  ( ) good (10)  Poor, but diagnostic  ( ) too dark (5)  ( ) too light (5)  ( ) unacceptable (0) |
| Graininess (20) | | | PP3 | ( ) no grain visible (20)  ( ) minimal grain (13)  ( ) grainy, but does not interfere with diagnosis (6)  ( ) grain interferes with diagnosis (0) |
| Detail (30) | | | PP4 | ( ) optimal (30)  ( ) good (20)  ( ) poor detail, but does not interferes with diagnosis (10)  ( ) lack of detail interferes with diagnosis (0) |

In the Lungman phantom study, the total average BRH scores of the V_new_ and V_con_ chest radiographic images were compared at the six radiation doses tested herein with the use of a box-whisker plot. According to the ALARA principle, to determine a dose reduction rate, image quality degradation was accounted from the reference image quality at the 100% dose. In other words, an achievable dose reduction did not compromise the reference image quality at the 100% dose. The achievable dose reduction was determined based on the analysis of the evaluated image quality scores with a measure of statistical significance between V_new_ and V_con_ at each dose level, with the use of Welch two sample t-tests. Interobserver agreement between the two radiologists was quantified based on the intraclass correlation coefficient (ICC). The ICC values were interpreted as follows: < 0.40, poor; 0.40–0.59, fair; 0.60–0.74, good; 0.75–1.00, excellent. Statistical analysis was performed using MedCalc (version 18.11.6, MedCalc Software) and IBM SPSS Statistics for Windows (version 21.0, IBM Corp., Armonk, NY, USA). A p value < 0.05 was considered statistically significant.

In the Duke phantom study, the quantitative measures of SNR and CNR with V_new_ were higher than those with V_con_ in both the high- and low-contrast areas for the tested dose range (Fig 1). The SNR and CNR of V_new_ were 2 to 2.2 times and 1.3 to 1.5 times higher than V_con_ for the given dose range, respectively, in the high- and low-contrast areas. In the high-contrast areas, both objective measures showed that the image quality with V_new_ at the lowest dose were comparable to that with V_con_ at the reference dose, thus demonstrating dose benefits from the use of V_new_. In the low-contrast area, the differences of the measures between V_con_ and V_new_ decreased as the dose decreased. This implies that the noise reduction algorithm may require a minimum dose level to work correctly.


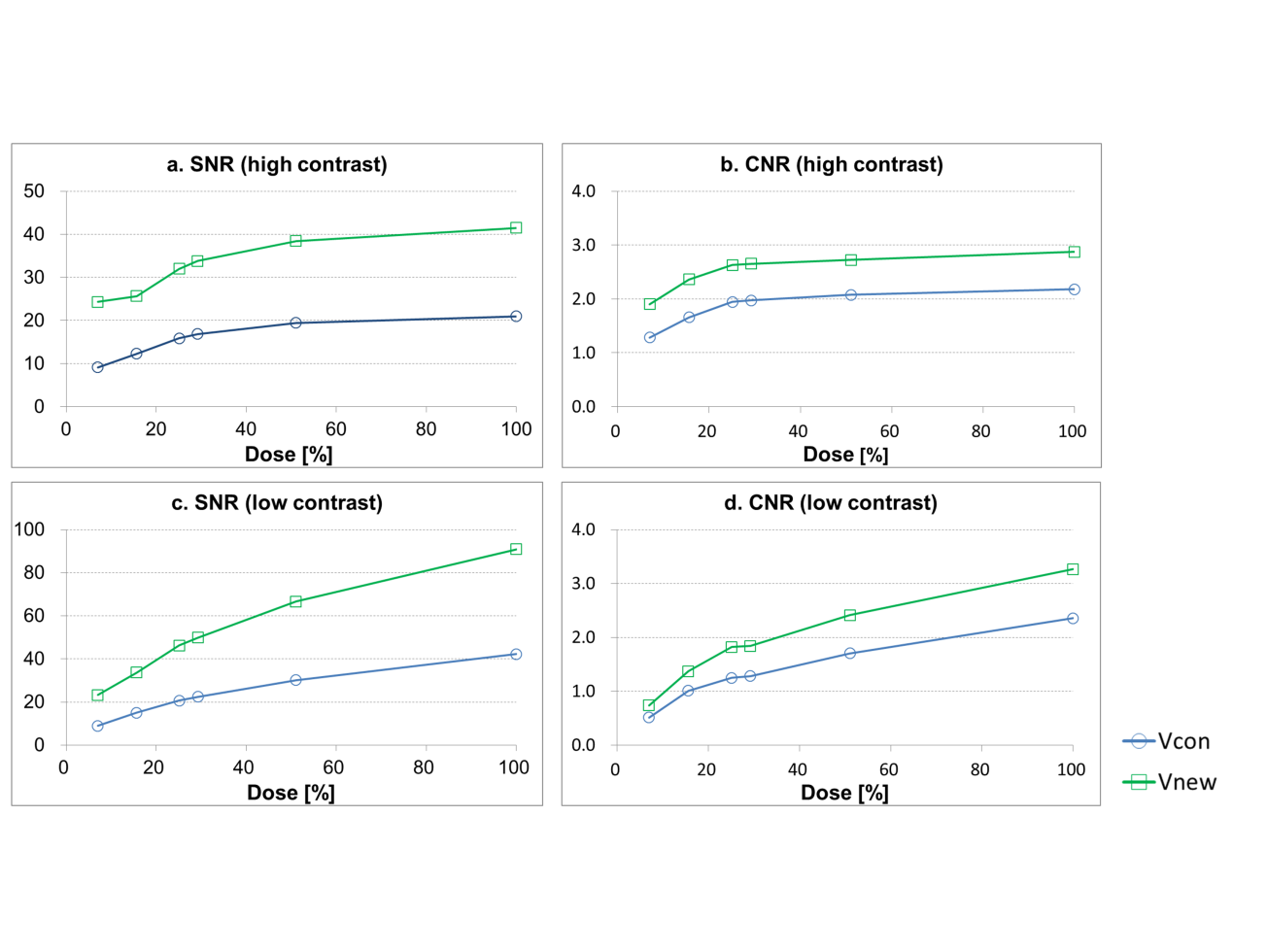


**Fig 1. Comparison of quantitative image analysis of V_con_ and V_new_ digital chest radiographs from the Duke phantom as a function of the radiation dose.** All graphs show the SNR and CNR values measured on (a, b) high- and (c, d) low-contrast areas of the V_con_ and V_new_ chest radiographs at the six radiation dose levels tested herein. The overall SNR and CNR values of V_new_ are higher than those of V_con_, which suggests that the performance of V_new_ can be better than that of V_con_ (SNR = signal-to-noise-ratio, CNR = contrast-to-noise ratio, ESE = estimated skin exposure).

Itemized evaluation scores were summarized in Table 2 based on the qualitative image analysis of the chest radiograph obtained with the Lungman phantom. Compared to the scores of the V_con_ images, the scores of the V_new_ images were equal or higher in all anatomical landmarks and for all physical parameters, for all the tested doses. Regarding the total BRH scores, in the V_new_ images, the lowest achievable dose was 25% of the reference dose and the image quality was not compromised, while in the V_con_ chest images, the lowest achievable dose was 51% the reference dose (Fig 2). Particularly, the total score difference between V_con_ and V_new_ images increased as the dose decreased. This was particularly evident at the lowest dose.

Table 2. Qualitative image analyses of V_con_ and V_new_ digital chest radiographs obtained with the Lungman phantom based on the modified BRH scoring system.

|  | Percentage of original dose | | | | | | | | | | | |
| --- | --- | --- | --- | --- | --- | --- | --- | --- | --- | --- | --- | --- |
|  | 100% | | 50% | | 30% | | 25% | | 15% | | 8% | |
|  | V_con_ | V_new_ | V_con_ | V_new_ | V_con_ | V_new_ | V_con_ | V_new_ | V_con_ | V_new_ | V_con_ | V_new_ |
| AL1 (5) | 5 | 5 | 5 | 5 | 4.5 | 5 | 4.5 | 5 | 3.5 | 5 | 3.25 | 4.75 |
| AL2 (5) | 5 | 5 | 4.5 | 5 | 4.25 | 4.75 | 3.75 | 4.50 | 2.75 | 4.25 | 2 | 3.75 |
| AL3 (15) | 15 | 15 | 15 | 15 | 9 | 15 | 7 | 15 | 6 | 9 | 5 | 9 |
| AL4 (15) | 15 | 15 | 15 | 15 | 15 | 15 | 13.75 | 15 | 13.75 | 15 | 12.5 | 15 |
| AL5 (15) | 15 | 15 | 15 | 15 | 15 | 15 | 15 | 15 | 15 | 15 | 15 | 15 |
| AL6 (30) | 30 | 30 | 30 | 30 | 30 | 30 | 30 | 30 | 30 | 30 | 30 | 30 |
| PP1 (35) | 35 | 35 | 35 | 35 | 35 | 35 | 32 | 35 | 26 | 32 | 26 | 32 |
| PP2 (15) | 13.75 | 15 | 13.75 | 15 | 13.75 | 15 | 12.5 | 15 | 10 | 15 | 10 | 13.75 |
| PP3 (20) | 20 | 20 | 20 | 20 | 16.5 | 18.25 | 11.25 | 18.25 | 9.5 | 14.75 | 4.75 | 7.75 |
| PP4 (30) | 30 | 30 | 30 | 30 | 27.5 | 30 | 22.5 | 30 | 12.5 | 27.5 | 5 | 15 |

V_con_ = Lungman phantom chest radiograph obtained with conventional algorithm

V_new_ = Lungman phantom chest radiograph obtained with new algorithm

BRH = Bureau of Radiological Health AL = Anatomic landmark PP = Physical parameter The numbers in parentheses in the first column indicate the perfect score of each item. The score of each item according to the radiation dose, is the average of the average scores of the two radiologists who evaluated every image twice.


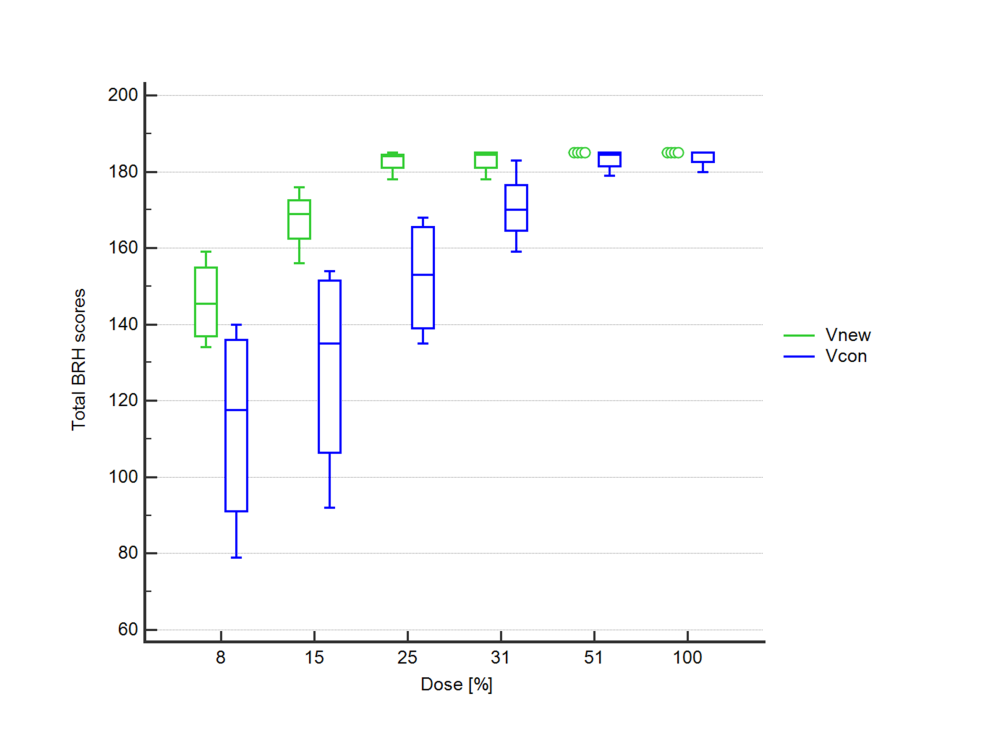


**Fig 2. Box–Whisker plots for total BRH scores of V_con_ and V_new_ digital chest radiographs obtained with the Lungman phantom as a function of the radiation dose.** The Box–Whisker plots of V_new_ and V_con_ images are similar at 51% and 100% of the original dose. However, the representative values (minimum, maximum, and median) of the Box–Whisker plot are reduced in the V_con_ images compared to those of the V_new_ images at all dose levels as dose decreases.

Table 3 summarizes the comparison of the total BRH scores between the V_con_ and V_new_ images using Welch two sample t-tests as a function of all the tested radiation doses. There were no significant differences in the total BRH scores between V_new_ images with 100%, 50%, 31%, and 25% of the original dose and V_con_ images with 100% and 50% of the original dose. Therefore, the quality of the V_new_ image acquired at 25% of the original dose was statistically similar to that acquired at 50% V_con_ of the original dose. This means that the new algorithm was able to reduce the administered doses by at least 50% compared to the doses used with the conventional method without compromises in image quality.

**Table 3. Comparison of total BRH scores between V_con_ and V_new_ digital chest radiographs obtained with Lungman phantom according to the radiation dose using Welch two sample t-tests.**

| **V_con_**  **V_new_** | **8%** | **15%** | **25%** | **31%** | **50%** | **100%** |
| --- | --- | --- | --- | --- | --- | --- |
| **8%** | - | p = 0.33  MD; 17  95% CI;  -26.1–60.1 | p = 0.54  MD; -6.3  95% CI;  -30.5–18 | p = 0.02  MD; -24.5  95% CI;  -42.8–6.2 | - | - |
| **15%** | - | - | p = 0.15  MD; 15.3  95% CI;  -8.4–38.9 | p = 0.66  MD; -3  95% CI;  -18.9–12.9 | p = 0.03  MD; -15.8  95% CI;  -28.4–3.1 | - |
| **25%** | - | - | - | p = 0.08  MD; 12.3  95% CI;  -2.7–27.2 | p = 0.82  MD; -0.5  95% CI;  -5.8–4.8 | p = 0.64  MD; -1  95% CI;  -6.0–4.0 |
| **31%** | - | - | - | - | p = 0.91  MD; -0.3  95% CI;  -5.7–5.2 | p = 0.73  MD; -0.8  95% CI;  -6–4.5 |
| **50%** | - | - | - | - | - | p = 0.39  MD; 1.3  95% CI;  -2.7–5.2 |
| **100%** | - | - | - | - | - | - |

MD = median difference CI = confidence interval

The calculated ICC values of the BRH scores were 0.8 or more in all the images obtained by both the V_con_ and V_new_ algorithms. These are considered to represent an excellent interobserver agreement (Fig 3). However, all ICC values in the V_new_ image were generally higher than those in the V_con_ image, and the interobserver agreement remained relatively constant in the V_new_ image, even when the dose decreased.


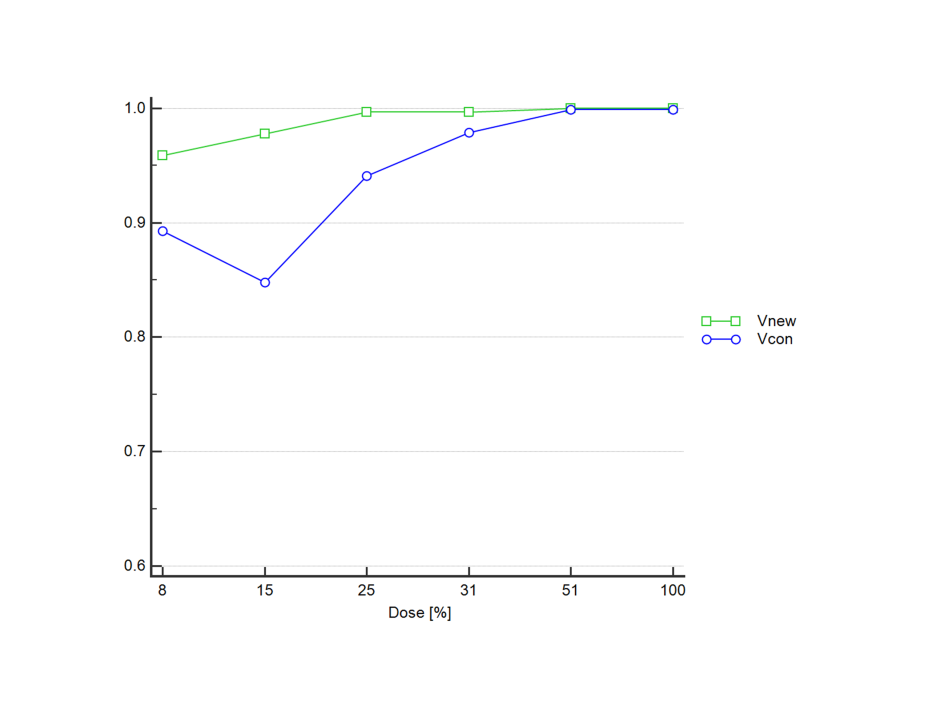


**Fig 3. Interobserver agreement on BRH scores of V_con_ and V_new_ digital chest radiographs obtained with the Lungman phantom according to the radiation dose.** The ICC value of the V_con_ and V_new_ images are almost the same at 51% and 100% of the original dose. However, as the dose decreases, the V_con_ images yield significantly lower ICCs compared to those of the V_new_ images.

1. **Summary**

This spatial noise reduction algorithm improved the overall image quality in low-dose chest radiographs of both semi-anatomical and anthropomorphic chest phantoms, even at extremely low doses. This can dramatically reduce the dose of digital chest radiography and overcome the current concerns on accumulated patient doses. However, although our preclinical experiment has been performed only on non-human phantoms, we have been able to approximate the minimum dose of radiation that can be applied to humans through our experiment. Based on these results, additional clinical studies could be expected to evaluate the normal anatomical structure and pathologic findings of humans.

**References**

1. Portilla J. Full blind denoising through noise covariance estimation using Gaussian scale mixtures in the wavelet domain. 2004 International Conference on Image Processing, 2004. ICIP'04. Vol. 2. 1217 - 20. doi: 10.1109/ICIP.2004.1419524.
2. Liu Y, Wang J, Chen X, Guo Y, Peng Q. A robust and fast non-local means algorithm for image denoising. Journal of Computer Science and Technology. 2008;23(2):270-9.
3. Matrecano M, Poggi G, Verdoliva L. Improved BM3D for Correlated Noise Removal. Proceedings of the International Conference on Computer Vision Theory and Applications (VISAPP-2012), 129-34.
4. Myers KJ, Barrett HH, Borgstrom MC, Patton DD, Seeley GW. Effect of noise correlation on detectability of disk signals in medical imaging. Journal of the Optical Society of America A. 1985;2(10):1752-9.
5. Cameron JR, Albert JA. Optimization of Chest Radiography: Proceedings of a Symposium Held at the University of Wisconsin, Madison, Wisconsin, April 30-May 2, 1979. Rockville, Md: U.S. Dept. of Health and Human Services, Public Health Service, Food and Drug Administration, Bureau of Radiological Health, 1980; 1980.
